# Fungal Composition and Diversity of the Tobacco Leaf Phyllosphere During Curing of Leaves

**DOI:** 10.3389/fmicb.2020.554051

**Published:** 2020-09-04

**Authors:** Qian-Li Chen, Lin Cai, Han-Cheng Wang, Liu-Ti Cai, Paul Goodwin, Jun Ma, Feng Wang, Zhong Li

**Affiliations:** ^1^Key Laboratory of Molecular Genetics, Guizhou Academy of Tobacco Science, Guiyang, China; ^2^College of Agriculture, Guizhou University, Guiyang, China; ^3^College of Plant Protection, Southwest University, Chongqing, China; ^4^School of Environmental Sciences, University of Guelph, Guelph, ON, Canada; ^5^Qianxinan Branch of Guizhou Tobacco Company, Guiyang, China

**Keywords:** high-throughput sequencing, fungal composition and diversity, *Rhizopus oryzae*, tobacco pole rot, flue-curing

## Abstract

*Rhizopus oryzae* causes tobacco pole rot in China during tobacco flue-curing. Flue-curing is a post-harvest process done to prepare tobacco leaves and involves three different stages: the yellowing stage has the lowest temperatures and highest humidity, then the color-fixing stage has higher temperatures and medium humidity, and finally the stem-drying stage has the highest temperatures and lowest humidity. In this study, fungal culturing and IonS5XL high-throughput sequencing techniques were used to reveal the fungal community of the petioles and lamina of tobacco leaves infected with pole rot during flue-curing. A total of 108 fungal isolates belonging to 6 genera were isolated on media. The most common fungal species isolated was the pathogen, *R. oryzae*, that was most often found equally on petioles and laminas in the color-fixing stage, followed by saprotrophs, mostly *Aspergillus* spp. High-throughput sequencing revealed saprotrophs with *Alternaria* being the most abundant genus, followed by *Phoma*, *Cercospora*, and *Aspergillus*, whereas *Rhizopus* was the tenth most abundant genus, which was mostly found on petioles at the yellowing stage. Both culturable fungal diversity and fungal sequence diversity was higher at stem-drying stage than the yellowing and color-fixing stages, and diversity was higher with leaf lamina than petioles revealing that the changes in fungal composition and diversity during the curing process were similar with both methods. This study demonstrates that the curing process affects the leaf microbiome of tobacco during the curing process, and future work could examine if any of these saprotrophic fungi detected during the curing of tobacco leaves may be potential biocontrol agents for with pole rot in curing chambers.

## Introduction

Tobacco *(Nicotiana tabacum* L.) is a leafy, annual, solanaceous plant grown commercially for its leaves. It is one of the most widely grown commercial non-food crop in the world (Liu et al., 2017). The leaves of tobacco are normally first harvested in commercial field and then flue-cured in a baking barn (Naidu, 2001). The purpose of flue-curing is to produce dried leaves of suitable physical properties and chemical composition (Burton et al., 1988; Morin et al., 2014). Leaf curing typically goes three stages, the yellowing stage with temperatures of <45°C and relative humidity over 75%, color-fixing stage with temperatures of 45−55°C and relative humidity over 35%, and stem-drying stage with temperatures of 55−70°C and relative humidity under 30% (Zheng et al., 2017). During the curing process, many pathogens can attack the plant causing lesions and rots of tobacco leaves, including bacteria such as *Erwinia carotovora* and *Bacillus polymyxa* (Spurr, 1980), and fungi such as *Aspergillus*, *Penicillium*, *Alternaria*, and *Cladosporium* (Welty et al., 1968).

Recently, the fungus *Rhizopus oryzae* has been associated with tobacco pole rot in China (Wang et al., 2016a; Pan et al., 2019). The fungus grows from the basal part of the midrib of the sessile leaf and spreads on the lamina initially forming white cottony mycelia fading from white to black as spores are produced. Under high humidity and warm temperatures during the leaf yellowing stage, the fungus can rot leaves within the first 48 h (Kortekamp et al., 2003). Spores of the pathogen remain viable during the high temperatures of the stem drying stage and thus survive from year to year in a flue-curing barn (Gu et al., 2018). Inoculum of the pathogen has been detected from fresh tobacco leaves collected in the field and also from the tools used in the curing chamber (Wang H. C. et al., 2017; Wang X. et al., 2017). In the last 5 years, tobacco pole rot was the most severe and most common disease happened during curing in flue-cured tobacco in China with losses reaching up to 100% (Cai et al., 2019). Additionally, *R. oryzae* has been used for enzyme production and fermentation (Huang et al., 2015; Yuzbashev et al., 2015), it is also a human disease (Ibrahim et al., 2010).

Many studies have examined the correlation between the microbiome and plant disease, showing that the microbiome could be disease suppressive or conducive (Luo et al., 2019; Raza et al., 2019). OTU (Operational Taxonomic Units) biodiversity analysis of phyllosphere microbiome has been reported for during several tobacco diseases, including black shank, brown spot and pole rot of tobacco (Chen et al., 2019; Xiang et al., 2019; Liu et al., 2020). However, the study of Chen et al. (2019) on pole rot only examined the microbial phyllosphere on tobacco tissues sampled at the end of stem-drying stage, and thus it is unknown if the same changes occur during the curing process where tobacco pole rot occurs.

Many methods have been used to study microbial composition and diversity. Cultural methods are normally easy to perform but are time consuming and can only detect <1% of microorganisms (Gong et al., 2016; Chen et al., 2018). Culture-independent methods, particularly high-throughput sequencing, are more sensitive and reliable for the identification of culturable and non-readily culturable microorganisms (Jayawardena et al., 2018; Mboowa et al., 2018). Chen et al. (2019) found the fungal community composition, relative abundance and dominant fungal taxon of each sample that with different pole rot level were all different. When at genus level for sample leaf lamina, the dominant genus was *Aspergillus*, *Myrothecium, Rhodotorula*, and *Fusarium*. For sample leaf petioles, dominant genus was *Aspergillus* and *Alternaria*.

Due to serious damage that can be caused by tobacco pole rot, it is important to know how the microbiome changes on tobacco leaves with pole rot during the curing process. In a curing chamber, the temperature is normally lower and relative humidity normally higher at greater heights. Therefore, tobacco leaf lamina and petioles at the three different curing height positions during the three curing stages were selected. The fungal composition and diversity of the tobacco leaf phyllosphere was analyzed both by cultural and high-throughput sequencing techniques. The results reveal changes in the fungi associated with leaves having pole rot in a curing chamber, providing a list of saprobes present naturally present during the curing process that could be examined in the future as antagonists for biocontrol of tobacco pole rot.

## Materials and Methods

### Environmental Conditions

Air relative humidity was measured by a hygroscope. Air, lamina and petiole temperatures were measured by a baking thermometer. Leaf lamina and petiole wetness was measured by a hygrometer recorder, and wind speed was measured by an anemoscope. Measurements were taken at 2.5 m, 1.75 m, and 1 m above ground level in the curing chamber at 42, 94, and 140 h post-harvest (hph).

### Sampling Sites and Sampling Strategy

In August 2018 in Guizhou province (26°36′N, 107°59′E) of China, one curing barn with pole rot disease was chosen for sampling. Leaves were collected from three different curing positions at 2.5 m (upper), 1.75 m (middle), and 1.0 m (lower) from ground level. Leaves of *N. tabacum* cultivar Yunyan 87 were harvested on 7th August from a commercial field and placed in a curing chamber starting 9th August. At yellowing (42 hph), color-fixing (94 hph), and stem-drying (140 hph) stages, 10 g of tobacco leaves at the upper, middle and lower curing positions were randomly sampled, and the petioles and leaf laminas were then separated. For coding samples, the letter A was used for petioles and B for lamina, which was followed by the number 1 for yellowing stage, 2 for color-fixing stage, and 3 for stem-drying stage, and then finally followed by 1 for upper, 2 for middle, and 3 for lower height positions. For example, A31 was petiole during the stem drying stage at the upper chamber position. Three biological repeats were conducted (Table 1). Leaf samples were immediately taken to the laboratory of Guizhou Academy of Tobacco Science at 4°C prior to culturing or −80°C prior to high-throughput sequencing.

**TABLE 1:** Environmental conditions for the tobacco samples collected from curing chamber.

Period (h)	Sample name	Chamber position (m)	Air relative humidity (%)	Air temperature (°C)	Wind speed (r/min)	Petiole wetness (%)	Lamina wetness (%)	Lamina temperature (°C)
Yellowing (42)	A11-B11	2.5	91.5	37	960	78	67	35
	A12-B12	1.75	87.5	37.5	960	75	65	35.5
	A13-B13	1	84	38	960	73	63	36
Color-fixing (94)	A21-B21	2.5	73	47	1450	55	49	45
	A22-B22	1.75	66	47.5	1450	48	43	45.5
	A23-B23	1	57	48	1450	40	35	46
Stem-drying (140)	A31-B31	2.5	22	67	960	20	14	65
	A32-B32	1.75	12.5	67.5	960	11	8	65.5
	A33-B33	1	8.5	68	960	5	4	66

### Isolation and Molecular Identification of Leaf Culturable Fungi

Culturable fungi were isolated by using the tissue separation method (Attitalla et al., 2010). Fungi were isolated on potato dextrose agar (PDA) containing 20 g glucose, 6 g potato powder, and 20 g agar per liter of distilled water, or alkyl ester agar (AEA) containing 5 g yeast extract, 6 g NaNO_3_, 1.5 g KH_2_PO_4_, 0.5 g KCl, 0.25 g MgSO_4_, 20 ml glycerin, and 20 g agar per liter of distilled water (Xu et al., 2015).

Necrotic petiole and lamina tissue pieces (5 × 5 mm) were stertilized with 70% ethanol for 30 s, then with 10% sodium hypochlorite (NaOCl) for 2 min, air dried and placed on both PDA and AEA plates. After 5 days at 28°C in the dark, hypha was transferred a new plate for purification. Fungi with different pigments, growth rates and morphologies were isolated from the plates. All purified fungi were stored on PDA slants at 4°C.

For molecular identification of each culturable isolate, the rDNA internal transcribed spacer region (ITS) was amplified and sequenced. PCR-amplification was conducted using primers ITS1F (5′-CTTGGTCATTTAGAGGAAGTAA-3′) and ITS4 (5′-TCCTCCGCTTATTGATATGC-3′). The amplified fragments were sequenced, and used as a query in a BLASTN search of the NCBI nr database^1^. Matches with identity values higher than 98% were used for the identification of the isolates.

### ITS Amplification and High-Throughput Sequencing

DNA of symptomatic petioles and laminas were separately extracted using FastDNA§Spin kit according to the manufacturer’s instructions (MP Biomedicals, Santa Ana, CA, United States), and eluted in a final volume of 80 μL. Quantity and quality of the DNA solution were assessed by agarose gel electrophoresis, and concentration and purity were assessed by NanoDrop ND-2000 (Thermo Fisher Scientific, Waltham, MA, United States).

Full-length ITS rDNA of the pure fungal isolates was PCR-amplified using primers ITS5 (5′-GGAAGTAAAAGTCGTAACAAGG-3′) and ITS2 (5′-GCTGCGTTCTTCATCGATGC-3′). PCRs were carried out in 30 μL reactions in triplicate, with each reaction tube containing 3 μL of each primer (2 μM), 2 μL of template DNA (1 ng/μL), and 2 × Phusion High-Fidelity PCR Master Mix with GC Buffer 15 μL. The following PCR condition was used: 98°C for 1min, 98°C 10 s, 50°C 30 s, and 72°C 30 s for 30 cycles, and a final extension of 72°C for 50 min. PCR products were subjected to electrophoresis on a 2% agarose gel subsequently, and the targeted fragment size (ITS 306 bp) was purified with Gene JET (Thermo Fisher Scientific, Waltham, MA, United States). Leaf fungal DNA samples were sequenced at the Novogene Bioinformatics Technology Co., Tianjin, China using 250 bp paired-end sequencing with an Ion S5 XL platform (Thermo Fisher Scientific, Waltham, MA, United States).

### High-Throughput Sequencing and Statistical Analysis

High-throughput sequencing was performed using the Ion S5 XL platform at Novogene Bioinformatics Technology Co., Beijing, China. The script from Novogene Corporation was used for clipping barcode and primer sequences by Cutadapt (V1.9.1^2^). The UPARSE pipeline (v7.0.1001^3^) was used to analyze operational taxonomic units (OTUs) and other biological information of the sequences obtained from each sample. The similarity was set to 97%. Species annotation was added to the representative OTU sequences. The community composition of each sample was counted at the kingdom, phylum, class, order, family, genus and species levels by Unit (v7.2^4^). Spearman’s correlation analysis was used to determine the relationships between environmental factor and the relative abundances of keystone species (Clarke, 2010). After obtaining the sequencing result and calculation of OTUs matrix, Qiime (V. 1.9.1) was used for full-sample similarity comparison to analyze the alpha-diversity and calculate the observed-species, Chao1, Shannon, Simpson, ACE, and Good’s-coverage indices. R software (Version 2.15.3) was used to draw a dilution curve, rank abundance curve, and species accumulation. Beta diversity on both weighted and unweighted unifrac were calculated by Qiime software (Version1.9.1). Sequences alignments for ITS locus were carried out using Muscle (Version 3.8.31) (Edgar, 2004). Phylogenetic analysis was conducted with FastTree 2 (Version1.9.1) (Price et al., 2010) with the Maximum Likelihood (ML) method. Principal Component Analysis (PCA) was displayed by WGCNA packages and ggplot2 package in R software (Version 1.9.1). Each OTU was assigned to a functional guild using the FUNGuild database.

## Results

### Environmental Conditions

During the curing process where tobacco pole rot occurs in the curing chamber, relative humidity declined from 84−91.5% to 8.5−22%, lamina wetness declined from 63−67% to 4−14%, and petiole wetness declined from 73−78% to 5−20% (Table 1). In contrast, air temperature increased from 37−38 to 67−68°C, and lamina temperature increased from 35−36 to 65−66°C. Wind speed was only higher during the color-fixing stage. This shows that tissues are drying with increasing temperatures over time. Position in the chamber was correlated with relative humidity, decreasing at progressively lower positions from 91.5 to 84%, 73 to 57%, and 22 to 8.5% in the yellowing, color-fixing and stem-drying curing stages, respectively. In contrast, air temperature increased at progressively lower positions from 37 to 38°C, 47 to 48°C, and 67 to 68°C in the yellowing, color-fixing and stem-drying curing stages, respectively.

### Culture-Based Fungal Diversity and Abundance

A total of 108 fungal isolates belonging to 6 genera were obtained by tissue isolation method (Table 2). The sequence of each isolate was deposited in NCBI GenBank. More species were isolated on PDA (*Rhizopus oryzae*, *Epicoccum* sp., *Diaporthe* sp., *Aspergillus* sp., *Alternaria* sp.) than on AEA (*R. oryzae*, *Epicoccum* sp., *Cladosporium tenuissimum*, *Aspergillus* sp.). The most abundant species was *R. oryzae* comprising 33 of the 54 isolates on PDA and 35 of the 54 isolates on AEA, followed by *Aspergillus* sp. with 18 of the isolates on PDA and 15 of the isolates on AEA. The rarest genera were *Epicoccum* sp., *Diaporthe* sp., *Alternaria* sp. and *C. tenuissimum*.

**TABLE 2:** Molecular identification of the total leaf fungi isolated from tobacco petioles and lamina in the baking chamber where tobacco pole rot occurred.

Stages	Media	No. of Species	Species	Strain code (GenBank No.)	Leaf-position		Curing - position		
					Petiole	Lamina	Up	Middle	Lower
Yellowing	AEA	2	*Rhizopus oryzae*	AA111(MN010558), AA112(MN010559), AB111(MN006416), AA121(MN010561), AA122(MN010562), AA123(MN013932), AB121(MN013934), AB122(MN006419), AB123(MN006420), AA131(MN010563), AA132(MN013933), AB132(MN006423), AB133(MN006424)	6	7	3	6	4
			*Aspergillus* sp.	AA113(MN010560), AB131(MN006422), AB112(MN006417), AB113(MN006418), AA133(MN010564)	2	3	3	0	2
	PDA	2	*Rhizopus oryzae*	PA122(MN010551), PA131(MN010553), PA132(MN010554), PA133(MN010555), PA111(MN010548), PA112(MN010549), PA113(MN013935), PB112(MN006654), PA123(MN010552), PB121(MN006656), PB122(MN006657), PB123(MN013936), PB131(MN010536), PB132(MN010537), PB133(MN010538),	8	7	4	5	6
			*Aspergillus* sp.	PB111(MN006653), PB113(MN006655), AA133(MN010564)	1	2	2	0	1
Color-fixing	AEA	4	*Rhizopus oryzae*	AB211(MN006425), AB223(MN006430), AB233(MN006433), AA232(MN006405)	1	3	1	1	2
			*Epicoccum* sp.	AB231(MN006431), AB232(MN006432)	0	2	0	0	2
			*Cladosporium tenuissimum*	AA212(MN010566), AB212(MN006426)	1	1	2	0	0
			*Aspergillus* sp.	AA231(MN006404), AA233(MN006406), AA211(MN010565), AB213(MN006427), AB221(MN006428), AB222(MN006429), AA213(MN006400), AA221(MN006401), AA222(MN006402), AA223(MN006403)	7	3	3	5	2
	PDA	5	*Rhizopus oryzae*	PB232(MN010546)	0	1	0	0	1
			*Epicoccum* sp.	PB231(MN010545)	0	1	0	0	1
			*Diaporthe* sp.	PA231(MN006635)	1	0	0	0	1
			*Aspergillus* sp.	PB211(MN010539), PA232(MN006636), PA212(MN010557), PA221(MN006632), PB233(MN010547), PA211(MN010556), PA213(MN006631), PB213(MN010541), PA222(MN006633), PA223(MN006634), PB221(MN010542), PB222(MN010543), PA233(MN006637), PB223(MN010544)	8	6	5	6	3
			*Alternaria* sp.	PB212(MN010540)	0	1	1	0	0
Stem-dring	AEA	1	*Rhizopus oryzae*	AA312(MN006408), AA313(MN006409), AB311(MK988567), AB312(MK988568), AB313(MK988569), AA322(MN006411), AA323(MN006412), AB321(MK988570), AB322(MK988571), AB323(MN006434), AA331(MN006413), AA332(MN006414), AA333(MN006415), AA321(MN006410), AB331(MN006435), AB332(MN006436), AB333(MN006437), AA311(MN006407),	9	9	6	6	6
	PDA	2	*Rhizopus oryzae*	PA311(MN006638), PA312(MN006639), PA313(MN006640), PB313(MN006667), PA321(MN006647), PA322(MN006648), PA323(MN006649), PA331(MN006650), PA332(MN006651), PA333(MN006652), PB311(MN006665), PB312(MN006666), PB321(MN006668), PB323(MN006670), PB331(MN006671), PB332(MN006672), PB333(MN006673)	9	8	6	5	6
			*Aspergillus* sp.	PB322(MN006669)	0	1	0	1	0

When analyzed for curing stage combining lamina and petiole samples, diversity was highest during the color-fixing curing stage with 6 genera, and lowest at the stem-drying curing stage with 2 genera (Table 2). The relative abundance (% of isolates of a species out of the total number of isolates) during the yellowing curing stage showed that *R. oryzae* was most abundant (12.04% on AEA and 13.89% on PDA), during the color-fixing stage showed that *Aspergillus* sp. (9.26% on AEA and 12.96% on PDA) were most abundant, and during the stem drying stage showed that *R. oryzae* (16.67% on AEA and 15.74% on PDA) was most abundant. Isolates of *C. tenuissimum* (1.85% on AEA and 0% on PDA), *Epicoccum* sp. (1.85% on AEA and 0.93% on PDA), and *Diaporthe* sp. (0.93% on AEA and 0% on PDA) were only found during the color-fixing curing stage.

When analyzed for tissue type combining curing stages, diversity was highest for lamina with 5 genera compared to petioles with 4 genera (Table 2). The relative abundance showed that *Aspergillus* sp. were higher in both petiole (8.33% on AEA and 8.33% on PDA) and lamina (5.56% on AEA and 8.33% on PDA), whereas *R. oryzae* was highest in both petiole (14.81% on AEA and 15.74% on PDA) and lamina (17.59% on AEA and 14.81% on PDA).

### Sequence-Based Fungal Diversity

**FIGURE 1:**
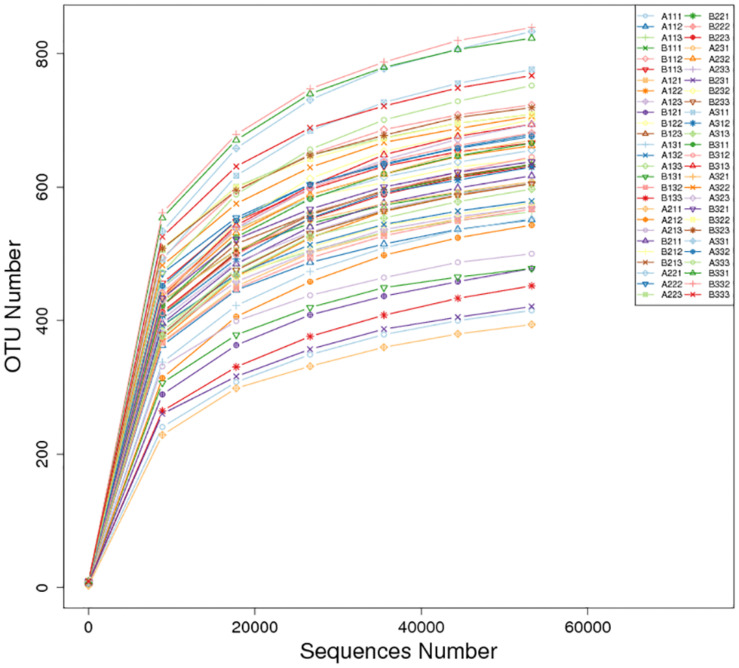
Rarefaction curves of OTUs across different tobacco leaf samples.

There was a total of 4,373,082 high-quality sequences across the 27 petiole and 27 lamina samples. A total of 2,238 OTUs at ≥97% nt identity were obtained from the 54 samples after the removal of low quality, chimeric and rare sequences resulting in an average number of sequences per sample of 80,983. The sequence of each sample was deposited in SRA database with accession PRJNA634435. When the number of sequences reached approximately 40,000, the rarefaction curves for all 54 samples revealed that they approached the plateau phase (Figure 1), suggesting that there was sufficient sequence coverage to describe the fungal composition.

Comparing between curing stages showed that the total number of OTUs progressively increased with significantly more OTUs at the stem-drying stage than the yellowing stage (Table 3). For petioles, the number OTUs increased between curing stages, with significant differences between the yellowing stage and the stem-drying stage. For leaf laminas, the number OTUs increased between curing stages, with significant differences between the yellowing stage and the stem-drying stage.

**TABLE 3:** Alpha- diversity indexes of fungal community and OTU numbers based on high-throughput sequencing in different samples.

Stage	Position	Sample	Petiole					Sample	Lamina				
			Shannon	Simpson	Chao1	ACE	OTU		Shannon	Simpson	Chao1	ACE	OTU
Yellowing	Up	A11	5.17 ± 0.77	0.92 ± 0.04	549 ± 58	557 ± 60	564 ± 66	B11	6.35 ± 0.06	0.96 ± 0.00	708 ± 45	707 ± 50	714 ± 51
	Middle	A12	5.42 ± 0.17	0.91 ± 0.01	643 ± 27	644 ± 23	634 ± 16	B12	5.50 ± 0.84	0.91 ± 0.69	614 ± 49	620 ± 48	622 ± 67
	Lower	A13	5.07 ± 0.73	0.86 ± 0.08	623 ± 21	639 ± 20	644 ± 34	B13	4.96 ± 0.64	0.88 ± 0.07	561 ± 58	567 ± 51	568 ± 50
	Average		5.22 ± 0.63	0.90 ± 0.06	604 ± 56	614 ± 56	608 ± 72		5.60 ± 0.83	0.92 ± 0.07	628 ± 79	613 ± 76.	635 ± 83
Color-fixing	Up	A21	3.24 ± 1.04	0.61 ± 0.20	522 ± 72	540 ± 73	537 ± 71	B21	5.44 ± 0.64	0.90 ± 0.08	678 ± 3	682 ± 6	695 ± 6
	Middle	A22	6.63 ± 0.33	0.97 ± 0.01	729 ± 18	732 ± 18	717 ± 35	B22	6.19 ± 0.28	0.96 ± 0.02	696 ± 24	707 ± 25	717 ± 16
	Lower	A23	6.35 ± 0.05	0.97 ± 0.00	675 ± 31	674 ± 28	678 ± 31	B23	5.66 ± 0.55	0.95 ± 0.02	622 ± 122	632 ± 118	645 ± 117
	Average		5.40 ± 1.66	0.85 ± 0.20	642 ± 99	649 ± 93	644 ± 92		5.67 ± 0.60	0.93 ± 0.50	666 ± 78	673 ± 76	686 ± 75
Stem-dring	Up	A31	5.75 ± 0.45	0.93 ± 0.04	715 ± 82	729 ± 79	745 ± 74	B31	5.63 ± 0.13	0.92 ± 0.02	730 ± 16	740 ± 14	736 ± 20
	Middle	A32	6.39 ± 0.11	0.97 ± 0.00	748 ± 2	760 ± 5	759 ± 19	B32	6.65 ± 0.19	0.97 ± 0.00	737 ± 38	737 ± 35	749 ± 25
	Lower	A33	6.34 ± 0.15	0.95 ± 0.01	821 ± 80	823 ± 77	824 ± 73	B33	6.89 ± 0.15	0.98 ± 0.01	855 ± 31	862 ± 31	879 ± 33
	Average		6.16 ± 0.40	0.95 ± 0.03	761 ± 80	771 ± 75	776 ± 70		6.39 ± 0.57	0.96 ± 0.03	774 ± 65	780 ± 65	788 ± 70
Total			5.60 ± 1.10	0.90 ± 0.12	670 ± 102	678 ± 100	676 ± 106		5.92 ± 0.75	0.94 ± 0.53	689 ± 95	695 ± 94	703 ± 99

*Richness and diversity estimation of the ITS sequencing libraries from the sequencing analysis. Shannon and Simpson are used to assess the community diversity, while Chao and Ace are used to evaluate the community richness. The values of mean ± SD of Three samples are shown in the table.*

Comparing between positions showed that during the yellowing stage, the number of OTUs for petioles were not significantly different, but the number of OTUs for leaf laminas were significantly greater in the upper position than the lower position (Table 3). During the color-fixing stage, there were significantly higher numbers of OTUs at the middle compared to the upper position for petioles, but no significant differences for leaf lamina. During the stem-drying stage, no significant differences were found for petioles based on position, but there were significantly lower OTU numbers in the upper and middle positions compared to the lower position for leaf lamina (Table 3).

Operational Taxonomic Units on petioles at the stem drying stage and on lamina at the stem drying stage, regardless of position, had highest diversity based on the average Shannon, Simpson, Chao1, and ACE values (Table 3). The next highest diversity of the OTUs was at the color-fixing stage for both petiole and lamina samples, regardless of position based on the average Shannon, Simpson, Chao1, and ACE values. The lowest diversity index values were found for OTUs at the yellowing stage for both petiole and lamina samples, regardless of position based on all four diversity indices.

### Taxonomic Composition of the OTUs

**FIGURE 2:**
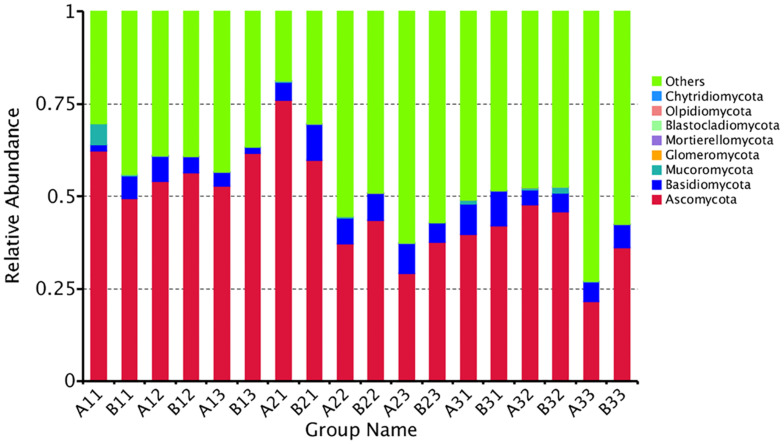
The relative abundance of different samples at Phylum level. Abundances of different bacterial phyla in the eighteen samples. The abundance was presented in terms of percentage among the total effective fungus sequences in each sample. The top ten abound taxa were shown.

The distribution of phyla for the OTUs showed that 88.29% of the clean sequence reads could be classified in the Ascomycota, Basidiomycota, Mucoromycota, Glomeromycota, Mortierellomycota, Blastocladiomycota, Olpidiomycota, and Chytridiomycota. Members of the other phyla were unassigned, and were likely not true fungi (Figure 2). The fungal communities were dominated by the Ascomycota (47.36%), followed by the Basidiomycota (5.88%), and the Mucoromycota (0.60%). Combined together, the Glomeromycota, Mortierellomycota, Blastocladiomycota, Olpidiomycota, and Chytridiomycota comprised only 0.01% of the reads.

Combining position and tissue type, the average number of reads per curing stage for the Ascomycota was 56.10%, 47.17%, 38.80% in the yellowing stage, color-fixing stage and stem-drying stage, respectively, indicating a decline as curing occurred. In contrast, the relative abundance for all samples for the Basidomycota was 4.12%, 7.11%, and 6.41% in the yellowing stage, color-fixing stage and stem-drying stage, respectively, indicating a peak in the color-fixing stage. The relative abundance for all samples for the Mucoromycota was 1.01%, 0.15%, 0.65% in the yellowing stage, color-fixing stage and stem-drying stage, respectively, indicating a decline during curing. Combining position and curing stage, the relative abundance of samples based on tissue type showed that Ascomycota were 46.68% and 48.03%, Basidiomycota were 5.57% and 6.18%, Mucoromycota were 0.96% and 0.24% for petioles and lamina, respectively, indicating that there was no significant difference based on tissue type.

**FIGURE 3:**
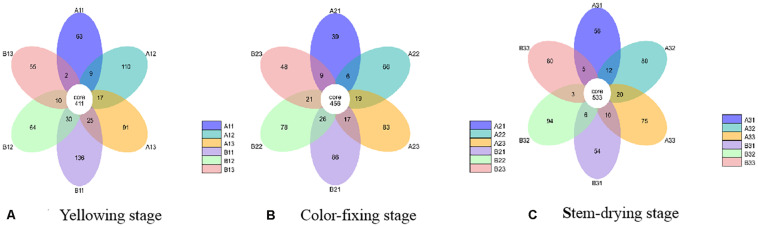
Venn diagram showing the number of fungal OTUs detected in different tobacco leaf tissues at three different curing stages. Panels **(A–C)** showing diagram at Yellowing, Color-fixing and Stem-drying stage, respectively. Numbers in the core section indicate shared OTUs for each sample at the curing stage. Numbers in the overlapping region indicate unique OTUs for the adjacent two samples. Numbers in the non-overlapping regions indicates unique OTUs for the sample.

At the OTU level, the differences between the communities from petiole and lamina samples at different curing stages were depicted with Venn diagrams. A total of 1023 OTU were discovered at yellowing stage, and 40.18% of them were shared OTU (Figure 3). Petiole samples from upper position and lamina samples from middle position contained more fungal varieties (602 and 547 OTU, respectively) than other four samples, as shown in Figure 3A. A total of 954 OTU were discovered at color-fixing stage, and 47.80% of them were shared OTU (Figure 3B). Petiole samples from upper position and lamina samples from down position contained more fungal varieties (585 and 575 OTU) than other four samples, as shown in Figure 3B. In comparison, a total of 1028 OTU were discovered at stem-dring stage, and 51.85% of them were shared OTU (Figure 3C). Petiole samples from middle position and lamina samples from middle position contained more fungal varieties (645 and 636 OTU, respectively) than other four samples, as shown in Figure 3C.

**FIGURE 4:**
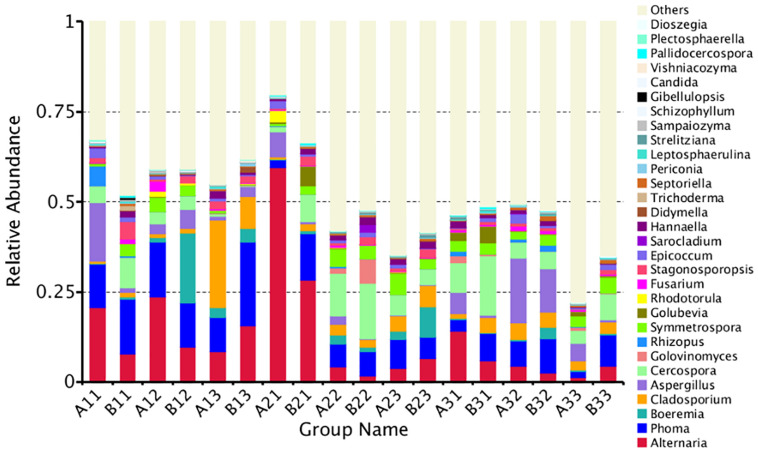
The relative abundance of different samples at genus level. Abundances of different bacterial genus in the eighteen samples. The abundance was presented in terms of percentage among the total effective fungus sequences in each sample. The top thirty abound taxa were shown.

A total of 35.97% of the OTUs could be classified at the genus level. The 30 most common genera are shown in Figure 4. Among those, the 10 highest number of reads were for *Alternaria*, *Phoma*, *Cercospora*, *Aspergillus*, *Cladosporium*, *Symmetrospora*, *Boeremia*, *Stagonosporopsis*, *Epicoccum*, and *Hannaella*. However, *Rhizopus*, which would include the pole rot pathogen, *R. oryzae*, had only 0.60% of the reads. Thus, the reads were dominated by OTUs for saprophytic, rather than pathogenic fungi.

**FIGURE 5:**
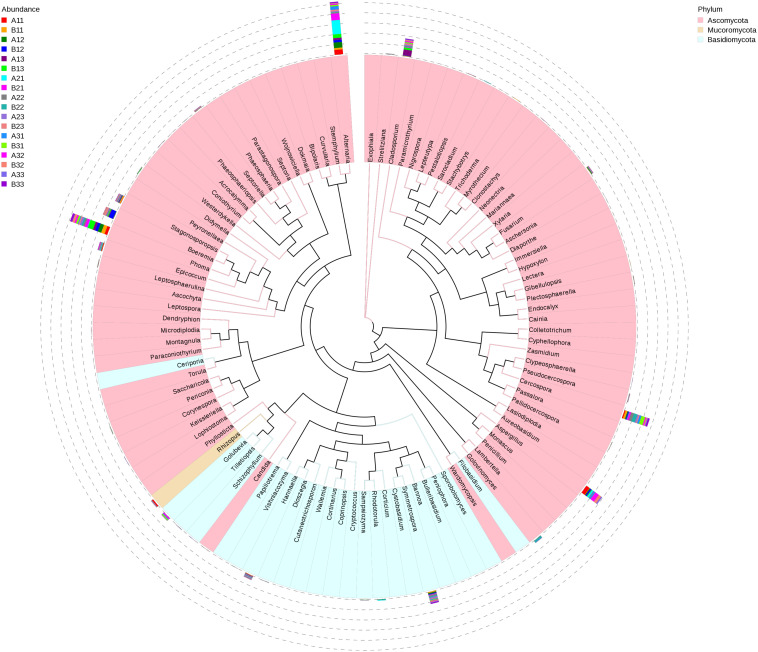
Maximum likelihood tree of 100 most abundant fungal genera in the Eighteen groups samples from the flue-cured tobacco obtained by analysis of ITS rDNA pyrosequencing data. Pink and Blue parts of the color range represented the genera of Ascomycota and Basidiomycota, respectively. A color-coded bar plot showed the distribution of each fungal genus in different samples.

A maximum likelihood tree of the 100 most abundant fungal genera showed that the most dominant fungi were in the Ascomycota, followed by Basidiomycota, and the least common fungi were in the Mucoromycota (Figure 5). For the Ascomycota, the dominant genera were *Alternaria*, *Aspergillus*, *Cerospora*, *Cladosporium*, *Phoma*, *Boeremia*, *Golovinomyces*, *Stagonosporopsis* and *Epicoccum.* For the Basidiomycota, the dominant genera were *Symmetrospora*, *Hannaella*, *Golubevia* and *Rhodotorula*. For the Mucoromycota, the dominant genera was *Rhizopus*, which would include *R. oryzae*.

The abundance of the top 10 genera varied considerably among the samples (Table 4). *Alternaria* was the highest at B21 followed by A31 and A21, indicating that it was more common in the upper position and somewhat more common in petiole than the lamina. *Phoma* was the highest at B13 followed by A12 and B11, indicating that it was also favored during the leaf yellowing stage. *Boeremia* was most common in B1, followed by B23 and B13, indicating that it has most abundant for lamina, curing stage or position. *Cercospora* was most abundant B31 followed by B22 and A22, indicating that it was more common in the upper position and somewhat more common in petiole than the lamina. *Aspergillus* was most common in A32, B32 and A33, indicating that the stem-drying stage at the middle and lower positions favored it. *Rhizopus* was the most dominant genus in A11, B32 and A31, indicating it was most common in petioles during the yellowing stage.

**TABLE 4:** The top10 dominant taxa and their relative abundance of fungal community of sample (Relative abundance %).

Stages	Leaf-position	Samples	*Alternaria*	*Phoma*	*Boeremia*	*Cladosporium*	*Aspergillus*	*Cercospora*	*Golovinomyces*	*Rhizopus*	*Symmetrospora*	*Golubevia*
Yellowing	Petiole	A11	20.80%	11.91%	0.23%	0.61%	16.33%	4.65%	0.01%	5.54%	0.63%	0.09%
		A12	23.56%	15.36%	1.27%	0.89%	2.95%	3.18%	0.04%	0.07%	3.98%	0.34%
		A13	8.55%	9.49%	2.70%	24.17%	1.14%	0.70%	0.04%	0.03%	0.86%	0.03%
	Lamina	B11	7.81%	15.24%	0.63%	1.33%	1.17%	8.50%	0.01%	0.30%	3.43%	0.11%
		B12	9.74%	12.26%	19.31%	1.39%	5.31%	3.78%	0.00%	0.04%	2.85%	0.09%
		B13	15.62%	23.24%	3.88%	8.80%	2.64%	0.41%	0.03%	0.05%	0.25%	0.02%
Color-fixing	Petiole	A21	59.59%	2.11%	0.24%	0.53%	7.07%	1.32%	0.02%	0.34%	0.61%	0.32%
		A22	4.17%	6.43%	2.40%	3.06%	2.31%	11.89%	1.42%	0.45%	4.91%	0.17%
		A23	3.81%	7.93%	2.48%	4.15%	0.18%	5.66%	0.06%	0.01%	5.94%	0.28%
	Lamina	B21	28.27%	12.88%	0.84%	1.92%	0.71%	7.46%	0.02%	0.10%	2.24%	5.55%
		B22	1.60%	6.81%	1.41%	2.07%	0.21%	15.35%	6.73%	0.00%	3.80%	0.08%
		B23	6.61%	5.78%	8.44%	6.08%	0.14%	4.46%	0.03%	0.00%	2.75%	0.10%
Stem-drying	Petiole	A31	14.14%	3.24%	0.46%	1.13%	5.86%	8.35%	1.87%	1.22%	3.00%	2.30%
		A32	4.45%	6.99%	0.43%	4.60%	17.94%	4.48%	0.06%	0.72%	2.17%	0.18%
		A33	1.30%	1.64%	0.40%	2.51%	4.94%	3.64%	0.80%	0.26%	2.91%	1.26%
	Lamina	B31	5.90%	7.61%	0.21%	4.24%	0.73%	16.46%	0.28%	0.03%	3.20%	4.58%
		B32	2.57%	9.54%	3.09%	4.15%	12.11%	4.80%	0.06%	1.60%	3.07%	0.10%
		B33	4.44%	8.66%	0.37%	3.26%	0.49%	7.27%	0.18%	0.05%	4.42%	0.43%
Total			12.38%	9.28%	2.71%	4.16%	4.57%	6.24%	0.65%	0.60%	2.84%	0.89%

### The Relationship to Environmental Parameters

**FIGURE 6:**
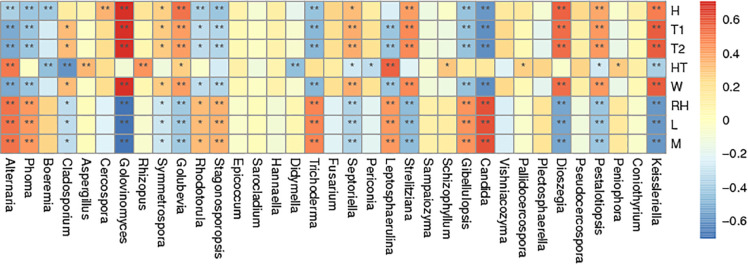
Spearman correlation analysis heatmap. Spearman’s correlations between major genus and environmental factor relative abundances. Baking time (H), wet-bulb temperature (T1), dry-bulb temperature (T2), height of tobacco hanging (HT), wind speed (W), room temperature (RH), leaf wetness (L), and petiole wetness (P). “*” and “**” indicated *p* < 0.05 and *p* < 0.01, respectively.

Spearman correlation analysis of the most abundant genera was made between air temperature, relative humidity, curing stage, position, wind speed and wetness of leaf and petiole (Figure 6). Air temperature significantly affected the abundance of *Golovinomyces.* Relative humidity significantly affected the abundance of *Alternaria*, *Phoma*, *Trichoderma*, *Leptosphaerulina*, *Gibellulopsis*, and *Candida*. Curing stage significantly affected the abundance of *Golovinomyces*, *Golubevia*, *Strelitziana*, *Dioszegia*, and *Pestalotiopsis*. Sample position significantly affected the abundance of *Alternaria*, *Aspergillus*, *Rhizopus* and *Leptosphaerulina*. Wind speed significantly affected the abundance of *Golovinomyces*, *Golubevia*, *Septoriella*, *Strelitziana*, *Dioszegia*, and *Pestalotiopsis*. Wetness of leaf and petiole significantly affected the abundance of *Alternaria*, *Stagonosporopsis*, *Trichoderma*, *Leptosphaerulina*, *Gibellulopsis* and *Candida*. In general, *Alternaria*, *Phoma*, *Golovinomyces*, *Strelitziana*, *Leptosphaerulina*, and *Pestalotiopsis* were the genera affected by the most environmental factors. *Rhizopus*, which would include the pole rot pathogen *R. oryzae* was the only one significantly affected by position.

### Spatial Distribution of Microbial Communities

**FIGURE 7:**
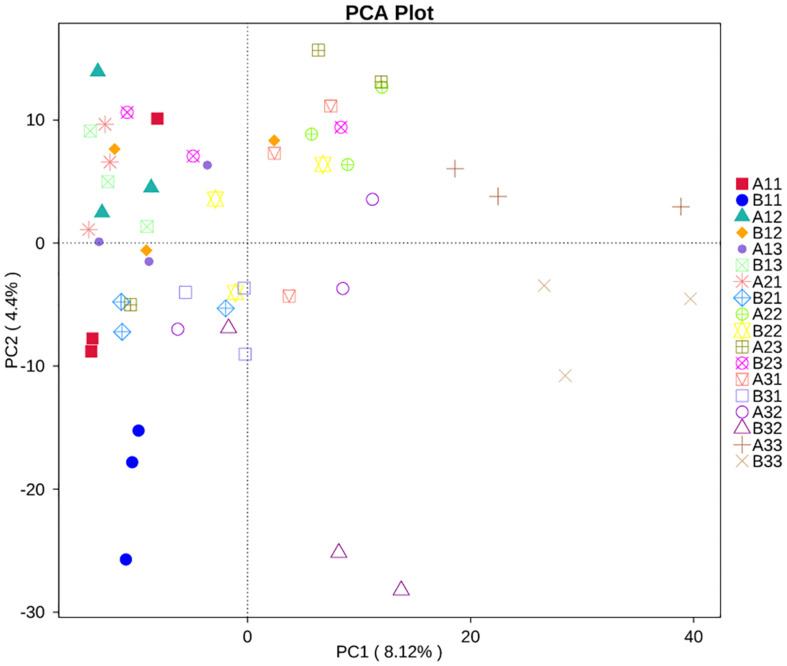
Principal Component Analysis (PCA) analysis of the fungal communities in the 18 groups samples.

Principal Component Analysis showed that the first two PCs accounted for 4.4% and 8.12% of the total variance in the fungal communities of the 18 sample groups (Figure 7). All of the fungal communities overlapped with each other, except for three distinctive fungal communities, which were for the upper petiole samples at the yellowing stage (B11), the lower lamina samples at stem-drying stage (B33) and the lower petiole samples (A33).

### Functional Guilds Analysis

**FIGURE 8:**
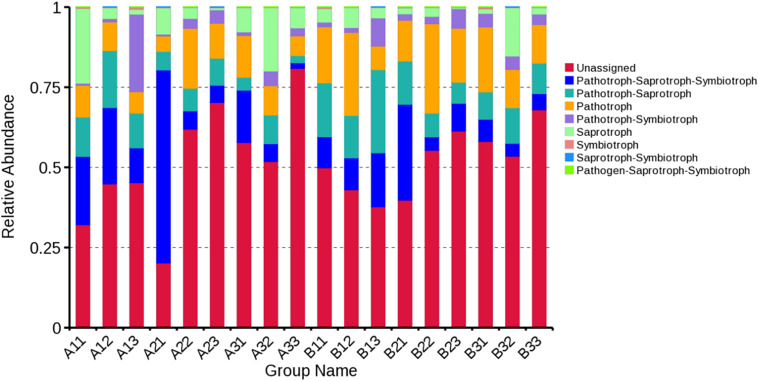
Relative abundance of fungal functional groups (guilds) based on OTU annotation table with disturbance frequency level.

FUNGuild database was used to classify the fungi in present study by ecological guild (Figure 8). Members of the pathotroph-saprotroph-symbiotroph were the most common at 13.68% of sequences, pathotroph was the second most common at 13.37% of sequences, pathotroph-saprotroph was the third most common at 10.56% of sequences, saprotroph was the fourth most common at 6.81% of sequences, pathotroph-symbiotroph was the fifth most common at 4.22% of sequences, and the least common were symbiotroph, saprotroph-symbiotroph and pathogen-saprotroph-symbiotroph at 0.20, 0.10, and <0.10% of the sequences, respectively. However, the unassigned sequences was the largest group at 51.77%. For the average based on sample type, pathotroph-saprotroph-symbiotroph was the most abundant in petioles and lamina. For the average based on curing stage, pathotroph was most abundant in leaf yellowing, color fixing and stem drying stages, respectively.

## Discussion

The diversity and function of tobacco leaf phyllosphere fungi were previously studied by culture-dependent methods (Lv et al., 2013), but the application of next generation sequencing technology to examine microbial populations in tobacco has revealed a much greater diversity of fungi (Zhao et al., 2007; Lei et al., 2017). In present study, a higher fungal diversity on tobacco leaves in a tobacco curing barn where tobacco pole rot occurred was found using high-throughput sequencing than a culture-dependent approach.

A comparison of cultivable fungal populations obtained on two different media (PDA and AEA) did not show any statistical differences, indicating that the media composition did not affect the detectable cultivable fungus strains. Only six culturable fungal genera were obtained in the present study, showing the limitations of cultivation-dependent methods. Microorganisms recovered from the environment by traditional culturing methods are rarely abundant in terms of their actual numbers or their functions in the environment, and it is estimated that less than 1% of microorganisms are readily culturable (Hugenholtz, 2002; Ma et al., 2017; Li et al., 2018). In this study, *R. oryzae* was the dominant culturable fungus in all samples, followed by *Aspergillus* sp. This finding agreed with some earlier reports that other fungi are associated with *R. oryzae* during barn rot in flue-cured tobacco (Cole, 1975; Zeng et al., 2014).

High-throughput sequencing showed that the Ascomycota was the dominant phylum followed by Basidiomycota and Mucoromycota. Ascomycota has been shown to be the most common phyllosphere fungi in many crops (Angelini et al., 2012), but both Ascomycota and Basidiomycota were the dominant taxons isolated from tobacco leaves (Wu et al., 2014). This is similar to this study. However, the taxons respond differently to the curing process. Ascomycota OTUs decreased greatly during curing, whereas Basidiomycota OTUs showed no significant changes during curing.

At a genus level, this study showed that the fungal OTUs were largely dominated by *Alternaria*, followed by *Phoma*, *Boeremia*, *Cladosporium*, *Aspergillus*, *Cercospora*, *Golovinomyces* and *Rhizopus*. In non-flue-cured and flue-cured tobacco leaves, the genera most frequently cultured, in decreasing frequency, were *Alternaria*, *Cladosporium*, *Epicoccum*, *Trichoderma*, *Nigrospora*, *Penicillium*, *Chaetomium*, *Fusarium* and *Aspergillus* (Welty et al., 1968; Welty and Lucas, 1969; Harvey, 1980; Nagrale et al., 2016). Thus, this study showed a considerable overlap in the frequency of fungal genera previously reported from tobacco leaves with *Alternaria* consistently being the most dominant leaf fungus. The high frequency of *Alternaria* is not surprising as it includes 100 species distributed worldwide over various agroclimtic zones and ecosystems and includes many important saprophytic and phytopathogenic fungi. *Alternaria alternata* is a facultative necrotrophic fungus on tobacco tissues producing toxins causing tobacco brown spot disease that commonly occurs during leaf senescence (Duan et al., 2010). In addition to detecting *Alternaria* OTUs, *Alternaria* sp. was isolated from leaves in this study indicating that brown spot disease was occurring in the curing chamber in this study. Although several studies, such as Welty and Lucas (1968), Welty et al. (1968) have found culturable *Penicillium* species being dominant on flue-cured tobacco leaves, relatively few *Penicillium* OTUs and no *Penicillium* isolates were found in this study.

With the proceeding of leaf curing in the curing chamber, all tobacco leaves have special fungal ecological niches. For all the fungi obtained from cultural method and the top 30 fungi gotten by high sequencing technique in this study, they either belongs to endophytes or to saprophytes. Most of them were plant pathogens and took higher abundance, including *Alternaria* (Wang et al., 2016b), *Phoma* (Yuan et al., 2016), *Cladosporium* (Wang et al., 2014), *Aspergillus* (Welty and Nelson, 1971), *Cercospora* (Newman and Townsend, 2016), *Golovinomyces* (Wang et al., 2012), *Rhizopus* (Wang et al., 2016a), *Stagonosporopsis* (Wang et al., 2018), and *Epicoccum* (Guo et al., 2020). These fungi are frequently reported to cause tobacco leaf disease. Beyond these pathogens, some endophytes were also obtained in this study by high-throughput sequencing, such as *Diaporthe* (Santos et al., 2016), *Rhodotorula* (Firrincieli et al., 2015), *Sarocladium* (El-Sayed et al., 2020), *Trichoderma* (Hosseyni-Moghaddam and Soltani, 2013), *Periconia* (Verma et al., 2011), *Fusarium* (*2009*), etc. They took lower abundance and normally do not infect tobacco leaves. All those endophytes and tobacco leaf fungal pathogens coexist in the same environment of tobacco leaves. The fungal composition and diversity of both endophytes and pathogens were all modified with the proceeding of leaf curing in the curing chamber.

As all tobacco leaf samples were collected in a curing chamber where tobacco pole rot disease occurred, it was expected that *R. oryzae* would be detected as it is the causal agent of pole rot of tobacco (Peng and Yi, 2007; Wang et al., 2016a). In this study, *Rhizopus* was detected from all samples by using both cultural-dependent method and high-throughput sequencing technique, and it was detected at all curing stages in both petioles and lamina and at all positions in the curing chamber. However, *Rhizopus* OTUs were most common in petiole during yellowing stage at the upper position, and least common in lamina during color fixing at the middle and lower position. Similarly, cultures of isolates of *R. oryzae* were most common during yellowing and stem-drying stages, and least common in color-fixing stage. Thus, it is common under these conditions but is affected by environmental and biological conditions. It was not surprising that it could be found in all curing stages as the fungus can survive at much high temperatures (Gayed, 1972; Wickes, 2013) than those recorded in this study.

Previous studies showed that higher temperature and increased humidity favored pole rot disease development, and infection was usually first seen in the butts of leaves, which corresponds to the petiole tissue in this study (Welty et al., 1968; Paddick and Turner, 1973; Deng et al., 2006). In this study, pole rot was more serious on leaves located near the upper position in the curing chamber compared to the middle and lower positions. Environmental monitoring in the study showed that position in the chamber affected temperature and humidity, and also affected fungal OTU composition, especially for *Rhizopus*. The other fungal OTUs in this study which showed the most similar impacts of the environment as *Rhizopus* were *Aspergillus*, *Pallidocercospora* and *Didymella*. One hypothesis from these results would be that changing curing conditions to decease temperature or humidity at the up position in curing chamber may increase the levels of certain saprobes on the leaf. Future work could test this to determine if this could affect microbial interactions affecting the levels of tobacco pole rot.

Using the FUNGuild database to assign fungal genera to functional guilds (Nguyen et al., 2016), most of the fungal OTUs in this study were identified as pathotroph-saprotroph-symbiotroph, followed by pathotroph-saprotroph and pathotroph. This indicated that a large number of the fungi on the tobacco phyllosphere during curing have potential plant pathogenic characteristics as well as many can be saprotrophs. This includes genera with species of known tobacco pathogens, such as the facultative necrotrophs, *A. alternata* that causes brown spot (Wang et al., 2016b), *Phoma omnivirens* that causes black spot stalk (Jiang et al., 2018) and *Cercospora nicotianae* that causes frogeye (Fajola and Alasoadura, 1973), as well as the obligate biotroph, *Golovinomyces* that causes powdery mildew (Mara et al., 2012). Most likely, these pathogens have arrived on tobacco leaves from diseased tissue in the field, and some could be growing both pathogenically inside the leaves as well as saprophytically on leaf surfaces during curing.

This study enlarges our knowledge of the fungal community of the tobacco phyllosphere on tobacco leaves during flue-curing. It showed that the environment during curing can impact fungal community composition and diversity, including fungal pathogens, such as those causing tobacco pole rot and brown spot. In addition to fungi, there are many bacteria associated with tobacco leaves, such as those able to cause leaf rot of tobacco (Spurr, 1980). More studies need to be conducted in this topic in the future. In this study, an air-rising curing chamber was used, but there are also air-falling chambers used for tobacco curing (He et al., 2017; Ngoni et al., 2017). It would interesting to learn in future studies if the two types of air flow systems would have different impact of microbial composition and diversity during tobacco leaf curing.

## Conclusion

In conclusion, the obtained data from this study showed that the leaf fungal communities at yellowing, color-fixing and stem-drying stages were markedly different in terms of alpha and beta diversity. *Alternaria*, *Phoma*, *Boeremia*, *Cladosporium*, *Aspergillus*, *Cercospora*, *Golovinomyces* and *Rhizopus* were the main fungal OTUs in the curing chamber, and as curing progressed, humidity and temperature were the key environmental factors shaping the leaf fungal community. Understanding the dynamics of the fungi associated with tobacco leaves during curing provides opportunities for future studies to manipulate those populations, either by culturing and applying saprophytic fungi identified in this study or by modifying curing conditions, such as increasing air speed to decrease humidity in the curing chamber or shortening the yellowing stage, in order to alter the levels of tobacco pole rot.

## Author’s Note

This manuscript has been released as a pre-print at Research Square (Chen et al., 2020).

## Data Availability Statement

The datasets presented in this study can be found in online repositories. The names of the repository/repositories and accession number(s) can be found in the article/supplementary material.

## Author Contributions

H-CW conceived and designed the experiments. Q-LC, LC, and L-TC performed the experiments. Q-LC and H-CW analyzed the data. Q-LC, H-CW, JM, FW, PG, and ZL wrote and revised the manuscript. All authors contributed to the article and approved the submitted version.

## Conflict of Interest

The authors declare that the research was conducted in the absence of any commercial or financial relationships that could be construed as a potential conflict of interest.

## Footnotes

1 http://www.ncbi.nlm.nih.gov/

2 http://cutadapt.readthedocs.io/en/stable/

3 http://www.drive5.com/uparse/

4 https://unite.ut.ee/
